# Unraveling the Spectrum: A Comprehensive Review of Autism Spectrum Disorder in India

**DOI:** 10.7759/cureus.62753

**Published:** 2024-06-20

**Authors:** Punam Uke, Sarika Gaikwad, Keta Vagha, Shailesh Wandile

**Affiliations:** 1 Pediatrics, Jawaharlal Nehru Medical College, Datta Meghe Institute of Higher Education and Reseach, Wardha, IND

**Keywords:** sensory integration therapy, diagnostic criteria, multidisciplinary, screening, autism spectrum disorder

## Abstract

Autism spectrum disorder (ASD) is a complex neurodevelopmental condition characterized by deficits in social interaction, communication difficulties, and repetitive behaviors that profoundly impact the lives of affected individuals and their families. This article provides a comprehensive overview of ASD, focusing on screening, diagnosis, and intervention strategies. Early signs of ASD can manifest in infancy, but parents may not recognize them until their child falls behind in meeting social milestones. This delay in recognition is often due to a lack of awareness, societal stigma, and limited knowledge about developmental and behavioral disorders.

Globally, ASD prevalence is increasing potentially due to broader diagnostic criteria, increased awareness, and improved screening practices. Screening for ASD is crucial for early identification and intervention. Various tools are available such as the Modified Checklist for Autism in Toddlers (M-CHATs), Trivandrum Autism Behavioral Checklist (TABC), and the Social Communication Questionnaire (SCQ). Diagnosing ASD involves using established criteria such as the Diagnostic and Statistical Manual of Mental Disorders, Fifth Edition (DSM-5), and specific diagnostic tools like the Autism Diagnostic Observation Schedule (ADOS) and the Indian Scale for the Assessment of Autism (ISAA).

Interventions for ASD should be multidisciplinary, involving professionals such as developmental pediatricians, psychologists, psychiatrists, special educators, occupational therapists, physiotherapists, speech therapists, and social workers. Applied behavior analysis (ABA), naturalistic developmental behavioral interventions (NDBIs), and parent-mediated treatment are among the evidence-based approaches. Additionally, speech-language therapy, motor therapy, and sensory integration therapy play vital roles in addressing the diverse needs of individuals with ASD. Medical interventions should be used alongside behavioral and environmental strategies.

Early screening, accurate diagnosis, and tailored interventions are essential for improving the lives of individuals with ASD. A multidisciplinary approach and increased awareness are crucial in addressing the growing prevalence of ASD worldwide.

## Introduction and background

**Figure 1:**
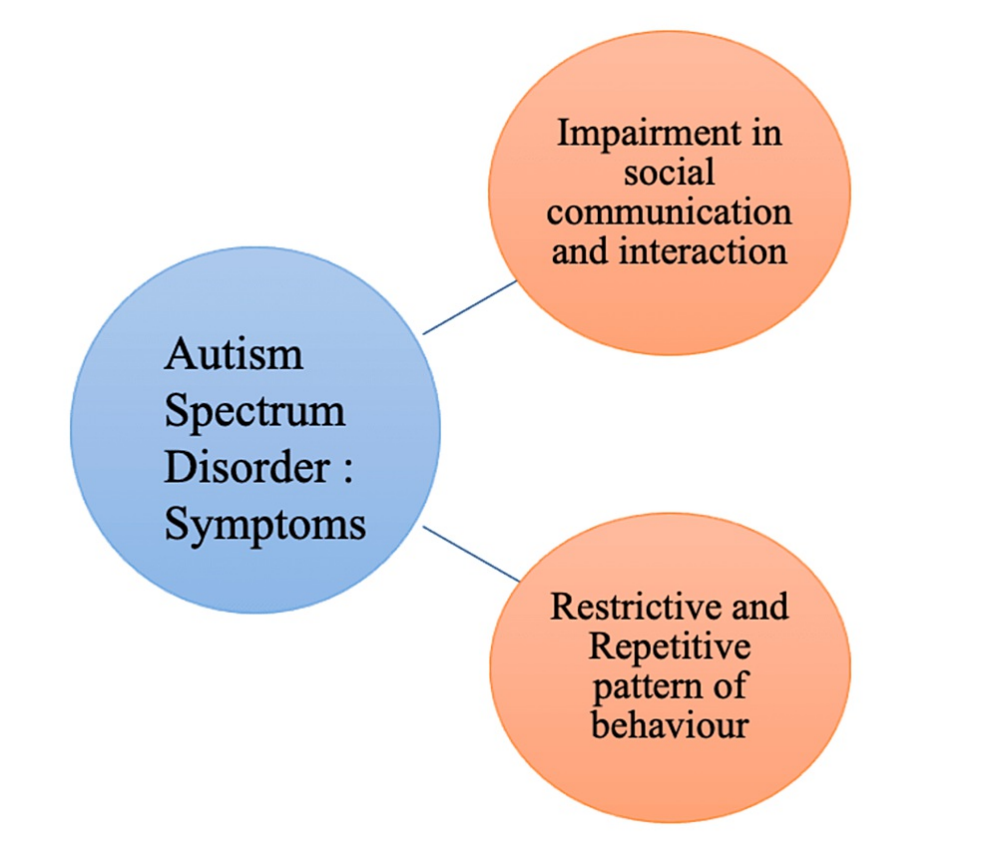
Two primary domains of autism spectrum disorder The author self-created the image

Autism spectrum disorder (ASD) is characterized by impairments in social communication, interaction, and restrictive, repetitive patterns of behaviors, interests, or activities [1]. Children with impairments in social communication and interaction do not interact with others, play alone, and do not participate in back-and-forth communication. They also have difficulty initiating and maintaining communication, along with difficulty in understanding nonverbal communication and gestures. These children lack pointing and joint attention. These children have restrictive and repetitive activities, along with fixed interests. The two primary domains that comprise the fundamental traits of autism are restricted, repetitive behavioral patterns and problems in initiating social communication and interaction, as seen in Figure 1.

It is a neurodevelopmental condition with major life-altering implications and high rates of medical and psychiatric comorbidities, which can be seen in up to 40% of children [2,3]. “Spectrum” in ASD indicates that each individual is affected in different ways, with mild-to-severe symptoms, often with overlapping comorbidities. Early signs of autism, such as lack of eye contact, can manifest as early as six months, but parents may not notice them until their child falls behind other youngsters of his or her age in meeting social needs [2]. When their child cannot verbally communicate in the early years, parents of autistic children grow increasingly concerned. Parents gradually notice social impairments as children become more ambulatory. Parents couldn’t notice signs and symptoms of ASD at an earlier age, mainly because of not being aware of this condition, profound ignorance, and social stigma in society regarding developmental and behavioral problems. Delay in diagnosis is also because of nuances in ASD presentation, which sometimes get missed by clinicians due to a lack of awareness among them. The absence of a fixed protocol in India for screening children also contributes to delayed ASD diagnosis. Since 2016, ASD has been included in disability certification under the Indian Rights of Persons with Disability Act.

Although there is no conclusive evidence to support the exact cause of autism, a multitude of studies conducted worldwide suggest that a number of chromosomal and genetic conditions like tuberous sclerosis and fragile X syndrome are attributed to this condition. Problems during delivery, perinatal complications, and elderly pregnancy have increased risk of ASD than children without these conditions. The Diagnostic and Statistical Manual of Mental Disorders, Fifth Edition (DSM-5) clubbed together all DSM-IV subgroups of the autistic spectrum, including pervasive developmental disorder (PDD) and Asperger syndrome, into a single entity ASD [1,4].

## Review

Epidemiology of ASD

ASDs were formerly thought to be a rare condition. But now 1 in 65 Indian children between the age group of two and nine are affected by ASD [4]. Up to 1.8-2 million children in India are thought to have ASD. According to a recent systematic analysis, the prevalence rate among children aged 0-17 in South Asian countries (Bangladesh, India, Sri Lanka) ranges from 0.09% to 1.07% for ASD [5]. ASD impacts about 5 million children in America, with a 1.7% estimated prevalence in 1 in 59 individuals [6]. Systematic epidemiological research is lacking in the field of autism in India, as compared to developed countries, because of which many cases go undiagnosed, resulting in a reduced prevalence rate than the actual value. Therefore, the cases that come to health facilities may represent only the tip of the iceberg, with many cases remaining hidden and undiagnosed.

The prevalence of ASD is increasing drastically all over the world, causing the societal impact of ASD [6-8]. ASD prevalence may be rising for a number of reasons, including expanding the diagnostic criteria through frequent, timely modifications of the DSM, along with PDD and other autistic conditions considered as single entities as ASD in recent times. Public awareness about the symptomology of the condition and increased practice of universal screening for ASD by health practitioners led to early and increased detection of children with autism [3]. Easily available facilities of early intervention and active school participation in providing school-based services for children with ASD might add to increased prevalence and improved outcomes [9].

Symptoms of ASD

ASD children have characteristic features, which are abnormal variations in normal social and ritualistic behavior for children of that age. ASD is presented with two main core features as per DSM-5, i.e., the deficit in social interaction/communication and restrictive, repetitive patterns of behavior [1]. Deficit in social communication/interaction can manifest as abnormalities in initiating and maintaining back-and-forth conversations, failing to respond to others by name, making very poor eye contact while speaking with them, using gestures in unusual ways, failing to understand imaginative play, and displaying very little interest in other kids [8].

Restrictive repetitive patterns of behavior can take the shape of difficulty with changes in routine, insistence on sameness in routine or food choices, rigid thinking patterns and fixation on unusual objects, repetitive body movements (such as spinning and flapping the hands), echolalia, and unusual processing of sensory stimuli from the auditory, visual, olfactory, and tactile senses [5]. Children with ASD frequently have co-morbid conditions, which have a severe negative impact on how well the child and family function, as well as on management [7]. Developmental or behavioral issues, such as anxiety, mood problems, attention-deficit hyperactivity disorder (ADHD), sleep issues, and seizures, are frequently present coexisting comorbidities with ASD [8]. Food refusal, constipation, self-injury, aggressiveness, and depression are additional prevalent issues [10].

Need for early screening for ASD

Many recent studies and research done all over the world have shown an increase in the prevalence of ASD worldwide, which emphasizes the importance of identifying children with clinical manifestations of ASD as early as possible during their developmental stages and implementing optimal early intervention [2]. The reliable age to identify and diagnose ASD could be as early as two years, as observable symptoms such as poor eye contact are present at this age [11]. Autism screening is a very effective standardized process to monitor children systematically and regularly for early signs of ASD. This ultimately leads to earlier diagnosis, which in the long term helps reduce delays and encourages early intervention to mitigate the disease burden in society [12]. Recent systematic studies conducted worldwide in different cultural settings have demonstrated that early intervention can improve outcomes, mainly in core features of ASD, IQ, language outcomes, and symptom severity [13,14]. The American Academy of Pediatrics (AAP) has recommended developmental screening in addition to universal screening for ASD during well-child visits at 18-24 months of age [15].

Screening tools for ASD

Screening tools are brief assessments based on small questions for identifying children at risk of neurodevelopmental disorders such as autism. Autism is a behavioral diagnosis, as all symptoms are atypical behavior patterns. It’s very important to mainly focus on autism-specific observable behaviors while developing a screening tool. Screening tools based on parents’ reports are easy to administer and require less professional assistance. However, close vigilance is needed while interpreting by a professional, specifically for first-time parents, as they may be less aware of appropriate developmental milestones and atypical patterns of behavior in their children. Some commonly used screening tools are listed in Table 1.

**Table 1:** Screening tools for ASD ASD: Autism spectrum disorder

Sr. No.	Name of screening tool	Age group for application	References
1	Modified Checklist for Autism in Toddlers (M-CHATs)	16 to 30 months	[16]
2	Trivandrum Autism Behavior Checklist (TABC)	2 to 6 years	[17]
3	Social Communication Questionnaire (SCQ)	4 years and above	[18]
4	Autism Behavior Checklist (ABC)	18 to 35 months	[19]
5	Ages and Stages Questionnaires (ASQs)	6 months to 5 years	[20]
6	Screening Tool for Autism in Toddlers (STATs)	24 to 35 months	[21]
7	Communication and Symbolic Behavior Scales Developmental Profile (CSBS DP)	6 to 24 months	[22]
8	Parents’ Evaluation of Developmental Status (PEDS)	Birth to 7 years and 11 months	[23]
9	Indian Autism Screening Questionnaire (IASQ)	3 to 18 years	[24]
10	Chandigarh Autism Screening Instrument (CASI)	1.5 to 10 years	[25]

Modified Checklist for Autism in Toddlers (M-CHATs)

The M-CHAT, revised with follow-up (M-CHAT R/F), is a free screening instrument that is accessible in numerous languages. The two-step parent questionnaire, which takes around 10 minutes to finish, consists of 20 closed-ended questions with yes/no answers, performed to screen children who are at high risk for ASD between the ages of 16 and 30 months [26]. When a child's score is higher than 8, it suggests that they might have ASD or other neurodevelopmental conditions. It's a two-step screening test: the first part is for parents, and the second part is for staff. A diagnostic evaluation should be ordered to confirm the diagnosis. Children who received a score of 3 to 7 should answer more interview questions about positive topics. Children who consistently receive positive scores on 3 to 7 items for ASD should be reevaluated for further diagnostic assessment. Scores below 3 are considered negative for ASD. The M-CHAT-R/F has a sensitivity of 96.6%, a specificity of 93.2%, and a satisfactory positive predictive value of 47.5%.

Trivandrum Autism Behavior Checklist (TABC)

TABC is a straightforward instrument created by the Child Development Center at the Medical College in Thiruvananthapuram, India, whose evaluation results were compared with the Childhood Autism Rating Scale (CARS) [27]. The four major domains of social interaction, communication, behavioral traits, and sensory integration are evaluated by the TABC. Responses include never (1), occasionally (2), frequently (3), and always (4). It also helps to classify autism according to severity, with scores of 20 to 35 denoting non-autism, 36 to 43 denoting mild to moderate autism, and 44 and higher denoting severe autism [28].

Social Communication Questionnaire (SCQ)

SCQ also known as the Autism Screening Questionnaire was derived from the Autism Diagnostic Interview-Revised (ADI-R). It’s available in many Indian languages and is often considered the gold-standard questionnaire used in many autism research studies [29]. The SCQ has 40 questions with answers in yes or no to be completed by parents. It is available in two forms: less than six years and more than six years. It takes less than 10 minutes for caregivers/parents to complete the test and less than a minute to score it.

Autism Behavior Checklist (ABC)

Children aged 18-35 months can benefit from the autism screening tool known as the ABC. It assesses 57 behaviors in five different domains: social, relational, body and object usage, language, and sensory. The checklist can be completed by parents or trained personnel, as they are fully aware of the many behaviors that children exhibit.

Ages and Stages Questionnaires (ASQs)

The parent or primary caregiver should fill out this general developmental screening tool. It consists of 19 age-related questions with a pass or fail response for 19 different developmental categories, comprising gross motor, fine motor, communication, problem-solving, and adaptation skills.

Screening Tool for Autism in Toddlers (STATs)

Designed for children who are at high risk for neurodevelopmental disorders such as autism, it is an activity-based screening tool. The administration of the assessment, which is done in 12 different tasks including play, communication, and imitation abilities, takes about 20 minutes. Children aged 24 to 35 months old can participate in it as an interactive session.

Communication and Symbolic Behavior Scales Developmental Profile (CSBS DP)

The CSBS DP was developed as a screening tool by Wetherby and Prizant. The infant/toddler checklist, which evaluates communication and symbolic behavior, is the first step in a routine. This test screens children in the age group of 6 to 24 months. If the first step doesn't work, the kid must assess communication with the other two CSBS DP components, namely the follow-up Caregiver Questionnaire and Behavior Sample (BS), based on the response.

Parents’ Evaluation of Developmental Status (PEDS)

The PEDS screening tool includes a 10-item questionnaire, with a primary focus on behavioral and developmental difficulties that parents must respond to. It helps to recognize children who present with early signs of autism from birth to seven years.

Indian Autism Screening Questionnaire (IASQ)

The Indian Scale for Assessment of Autism (ISAA), a frequently used diagnostic test for autism in India, served as the basis for the IASQ. This 10-item questionnaire has a yes/no response format and requires little training to use, making it crucial in areas with few medical services.

Chandigarh Autism Screening Instrument (CASI)

The CASI is specifically designed for community screening with the help of health workers. It is a 37-item questionnaire-based tool for children between 1.5 and 10 years.

Diagnostic tools for ASD

Children who are positive or at high risk in screening tools should undergo evaluation for diagnostic tools for the confirmation of diagnosis. Different diagnostic tools are available, but DSM-5 criteria is an easy and comprehensive diagnostic tool for initial diagnosis by general pediatricians and child psychologists [30]. A formal assessment of hearing, vision, and cognitive skills, along with a complete physical examination, is of crucial importance before the diagnostic assessment of ASD. Some commonly used screening tools are listed in Table 2.

**Table 2:** Diagnostic Tools for ASD ASD: Autism spectrum disorder; INCLEN: International Clinical Epidemiology Network

Sr. No.	Name of diagnostic tool	Age group for application	References
1.	Diagnostic and Statistical Manual of Mental Disorders, Fifth Edition (DSM-5)	Above 1 year	[1]
2.	Autism Diagnostic Observation Schedule (ADOS)	Above 1 year	[31]
3.	Indian Scale for the Assessment of Autism (ISAA)	3-9 years	[32]
4.	INCLEN Diagnostic Tool for Autism Spectrum Disorder (INDT-ASD)	2-9 years	[33]
5.	Childhood Autism Rating Scale (CARS)	2 years and above	[34]
6.	Autism Diagnostic Interview-Revised (ADI-R)	2 years and above	[35]
7.	Gillian Autism Rating Scale (GARS)	3-22 years	[36]

DSM-5

The basic symptoms in the DSM-5 were divided into two categories: restricted, repetitive behavioral patterns, and deficits in social communication and interaction. The DSM-5 requires that two of the four symptoms linked to restrictive and repetitive behaviors, along with all three symptoms of social communication deficits, be present in order for the diagnosis of ASD to be made [1]. The DSM-5 also helps in grading the severity of ASD. It is convenient and easy for pediatricians as well as for child psychologists so that a child can be started on early intervention at the earliest. The sensitivity of the DSM-5 criteria for ASD is generally high, ranging from 0.80 to 0.94, with specificity ranging from 0.79 to 0.98. The DSM‐5 Text Revision (DSM‐5‐TR) is the first published revision of DSM‐5 since its original publication in 2013 [30].

Autism Diagnostic Observation Schedule (ADOS)

The ADOS was the first tool for evaluating ASDs that was standardized and performance-based. A series of 'planned social occasions' that provide the kids an opportunity to react to various social situations aids in the evaluation of social play and communication. There are four 30-minute segments of the ADOS. Every module is made to evaluate the kid at the proper stage of language and development, such as one model for children who are pre-verbal or who have limited language skills, and another for children who are not verbally fluent. The ADOS Toddler Module (ADOS-2) helps to diagnose children as early as 12 months of age [37]. Sensitivity values for the ADOS-2 have been reported to range from 0.70 to 0.89, with specificity ranging from 0.69 to 0.91.

ISAA

There wasn't previously a scale that was suitable for use in an Indian context because the bulk of scales were developed in Western countries [38]. The National Institute for the Mentally Handicapped developed the ISAA. The ISAA includes screening questions on behavioral patterns, sensory and cognitive abilities, emotional receptivity, speech, and language communication, as well as social interaction and reciprocity. It takes 45 to 60 minutes to administer and requires training. It is a comprehensive 40-item tool that relies on information from parents and a child's observation. It can be used for follow-up and certification but not for screening in population research [32]. The ISAA score ranges from 40 to 200, and as the number rises, so does the severity of the disorders.

*International Clinical Epidemiology Network* (*INCLEN) Diagnostic Tool for Autism Spectrum Disorder (INDT-ASD)*

Diagnostic tool for ASD developed using DSM-5 criteria created by AIIMS (INDT-ASD). It can diagnose ASD with a 91.7% specificity and a 98.4% sensitivity [33]. There are two sections to the tool: A2 (2a, 2b, 2c, 2d) covers symptoms associated with restrictive repetitive behavior, which is one of the two core domains of the DSM-5. Section A1 (1a, 1b, 1c) covers items related to the social communication deficit type of symptoms of ASD. The process of scoring and administering the test takes about 45 to 60 minutes. In terms of play and peer interaction, it considers the wide range of ethnic and religious diversity that exists in this nation, together with its dynamic culture. It is intended for use by qualified personnel and is predicated on the history provided by main caregivers, as well as firsthand observation of a kid between the ages of two and nine.

CARS

The first CARS test was criticized for not being able to reliably identify higher-functioning autistic individuals because it was developed mostly by people with concurrent intellectual functioning [34]. An improved version of the CARS is called the CARS-2. It is the behavioral rating scale that is most frequently used for kids who are older than two. The CARS-2, now called the CARS2-ST for "Standard Form," is used with children who are predicted to have an IQ of 79 or lower and are less than six years old.

A special grading system known as the CARS2-HF for 'High Functioning' was developed for kids above six years old with an approximate IQ of 80 or more and clear communication. The CARS2-QPC (Questionnaire of Parent Concerns), an ungraded form, allows parents to record observations and gather information. The 15th category of the CARS, which consists of 15 categories, assesses how people generally perceive autism. The first 14 domains evaluate autism-related characteristics like verbal and nonverbal communication, as well as repetitive behaviors.

The severity of the impairment is indicated by a number between one and four assigned to each domain. Total scores can vary from 15 to 60; values less than 30 can be taken as within the normal range, values between 30 and 36.5% are considered to be mild to moderate autism, and values between 37 and 60% are considered to be severe autism.

ADI-R

Children, as well as adults, with autism can be identified using a diagnostic method that emphasizes behavior in three key domains: restricted and repetitive, stereotyped patterns and behavior; communication and language; and reciprocal social contact. The ADI-R is a very useful tool with mental ages of at least 18 months in both adults and children. This measure includes a standardized, in-depth parent interview lasting one and a half hours for parents of three- to four-year-olds, and three hours for older children [39]. Sensitivity and specificity values for the ADI-R have been reported to range from 0.70 to 0.90.

Gillian Autism Rating Scale (GARS)

By using this scale, schools, parents, and medical professionals can detect and diagnose autistic children between the ages of 3 and 22. It also assigns an ASD severity rating. It is a 42-item norm-referenced screening tool that includes a developmental history and collects data on certain traits frequently seen in kids with ASDs in three domains (stereotypical behaviors, communication, and social interaction).

Different interventions for ASD

**Figure 2:**
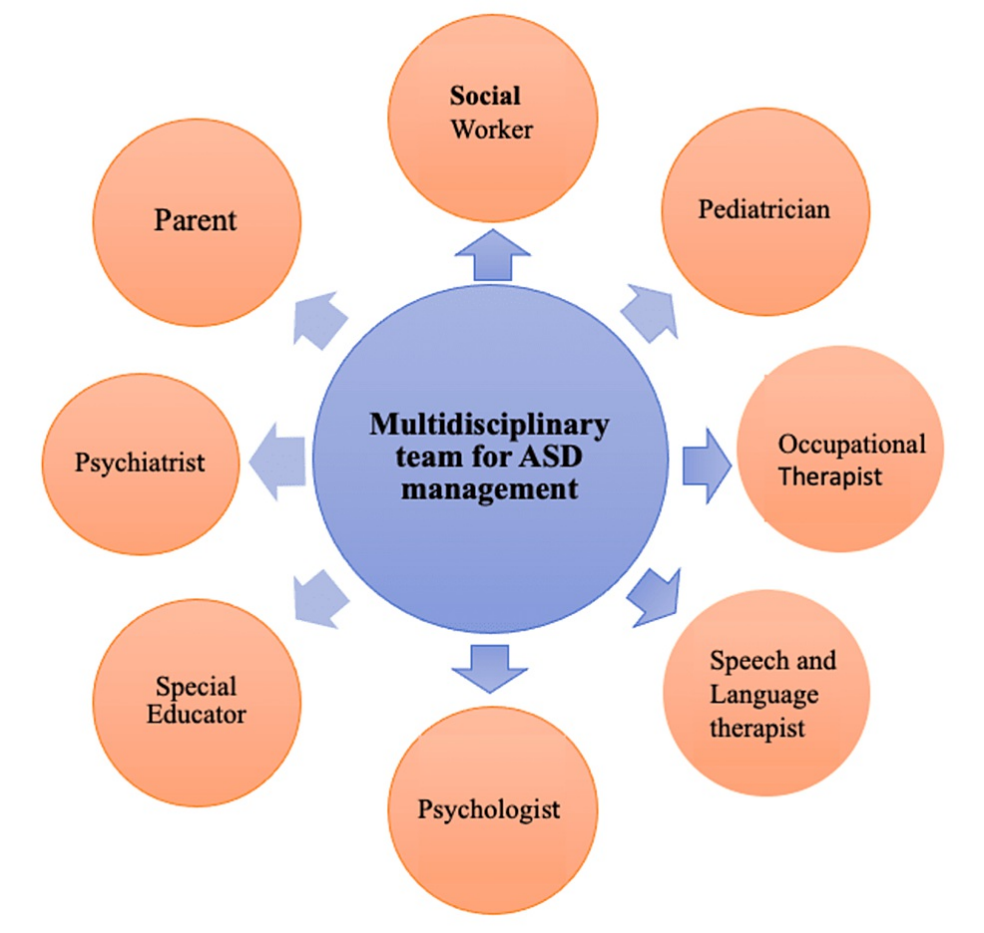
Multidisciplinary approach managing ASD Image credit: Dr. Punam Uke ASD: Autism spectrum disorder

Because of the complexity of autism, the assessment and management should be done with the help of a multidisciplinary team, which consists of a developmental pediatrician, psychiatrist, psychologist, audiologist, occupational therapist, speech therapist, special educator, and social worker, depicted in Figure 2.

Applied Behavior Analysis (ABA)

ABA is a set of principles that form the basis for many behavioral treatments. It is based on the science of learning and behavior. ABA is the science in which procedures derived from the principles of behavior are systematically applied to improve socially significant behaviors, which are activities important for daily life [40]. ABA significantly alters socially significant behaviors, resulting in improved behavior. It teaches skills to replace problem behaviors and increase positive behavior. Treatments utilizing ABA may concentrate on improving already present skills, such as social involvement, or minimizing harmful behaviors like self-abusive behavior that might obstruct a child's development [41]. ABA skills training programs, such as discrete trial training and incidental teaching, can require several hours each day.

Naturalistic Developmental Behavioral Interventions (NDBIs)

ABA and developmental concepts are used in NDBIs, which provide therapy in the context of naturally available social activities in natural environments. Some NDBIs use developmental sequences to guide goal development that is individualized to each child such as Early Start Denver Model (ESDM), Social Communication, Emotional Regulation, and Transactional Support (SCERTS). These therapies emphasize essential social learning abilities and learning objectives that are based on developmental stages [42].

Parent-Mediated Treatment

Several studies have demonstrated the potential benefits of focused interventions, such as social skills and activities of daily living provided by skilled caregivers like parents, within a therapeutic program [41]. This is especially useful in low-income countries where facilities for special educational centers are limited. Utilizing the Treatment and Education of Autism and Related Communication Handicapped Children (TEACCH) curriculum, along with an individually designed program modified to meet the goals of the Individualized Education Program (IEP), and actively involving parents, helps achieve effective outcomes [43].

Speech-Language Therapy

Speech-language therapy is the intervention that is most commonly used with children who have ASD [44]. Children who struggle with conversational skills may benefit from using Augmentative and Alternative Communication (AAC), which includes sign language, the Picture Exchange Communication System (PECS), and speech-generating equipment [45].

Motor Therapy

Children diagnosed with ASD may experience dystonia and difficulty coordinating. It is advised to employ occupational therapy services to assist fine motor and adaptive skills, including handwriting, handling toys, and self-care. Children with ASD frequently toe walk, and they benefit greatly from passive stretching, orthotics, and casting [46].

Sensory Integration Therapy

Sensory integration therapy for children with developmental and behavioral disorders offers clinicians crucial baseline knowledge and suggestions regarding common sensory-based therapies, including skin brushing, wearing weighted vests for proprioceptive stimulation, or engaging in kinesthetic stimulation. Play and sensory activities help children to improve sensory responses [47].

Medical Interventions

Always utilize medication in conjunction with the proper behavioral and environmental interventions. For the treatment of co-morbid psychiatric or neurodevelopmental problems, as well as behavioral symptoms that impair the child's ability to function on a daily basis, pharmaceutical therapy may also be necessary. Medications such as risperidone, aripiprazole (for maladaptive behaviors), methylphenidate, atomoxetine, clonidine, guanfacine (for ADHD), fluoxetine (for repetitive behaviors and rigidity), melatonin, and serotonin (for sleep disturbances) are some of the frequently used options [48]. 

## Conclusions

ASD is a neurodevelopmental condition characterized by a wide range of symptoms, mainly causing difficulties in social communication and restrictive and repetitive activities, and often accompanied by other comorbid conditions in children. These diverse behavioral challenges can create adjustment issues for both the child and their family within society. Early screening and accurate diagnosis are crucial, as they play a pivotal role in determining the future outcomes for these children. Managing ASD requires a multidisciplinary approach involving professionals from various fields to provide comprehensive care to each child's specific needs.
